# Tafamidis decreased cardiac amyloidosis deposition in patients with Ala97Ser hereditary transthyretin cardiomyopathy: a 12-month follow-up cohort study

**DOI:** 10.1186/s13023-023-02824-0

**Published:** 2023-09-13

**Authors:** Cheng-Hsuan Tsai, Chi-Chao Chao, Sung-Tsang Hsieh, An-Li Yu, Yuan-Kun (Aden) Wu, Mei-Fang Cheng, Ming-Jen Lee, Chia-Hung Chou, Chia-Tung Shun, Hsueh-Wen Hsueh, Jimmy Jyh-Ming Juang, Ping-Huei Tseng, Mao-Yuan Su, Yen-Hung Lin

**Affiliations:** 1 National Taiwan University College of Medicine, Graduate Institute of Clinical Medicine, Taipei, Taiwan; 2 Department of Internal Medicine, National Taiwan University Hospital and National Taiwan University College of Medicine, Taipei, Taiwan; 3 Department of Neurology, National Taiwan University Hospital, Taipei, Taiwan; 4 Division of Cardiology, National Taiwan University Hospital, Taipei, Taiwan; 5 5Cardiovascular Center, National Taiwan University Hospital, Taipei, Taiwan; 6 Department of Nuclear Medicine, National Taiwan University Hospital and National Taiwan University College of Medicine, Taipei, Taiwan; 7 Institute of Environmental and Occupational Health Sciences, National Taiwan University, Taipei, Taiwan; 8 Department of Obstetrics and Gynecology, Department of Internal Medicine, National Taiwan University Hospital and National Taiwan University College of Medicine, Taipei, Taiwan; 9 Department of Forensic Medicine and Pathology, National Taiwan University Hospital, Taipei, Taiwan; 10 Department of Pathology, Good Liver Clinic, Taipei, Taiwan; 11 Division of Gastroenterology, Department of Internal Medicine, National Taiwan University Hospital, Taipei, Taiwan; 12 Department of Medical Imaging, National Taiwan University Hospital, Taipei, Taiwan; 13 Department of Medical Imaging and Radiological Technology, Yuanpei University of Medical Technology, Hsinchu, Taiwan

**Keywords:** Cardiac magnetic resonance, Extracellular volume, Hereditary transthyretin amyloidosis, Tafamidis

## Abstract

**Background:**

Transthyretin cardiac cardiomyopathy (ATTR-CM) is a rare but life-threatening disease. Tafamidis is an effective treatment for patients with ATTR-CM, however its long-term effects on cardiac remodeling and cardiac amyloid deposition are unknown. This study aimed to used cardiac magnetic resonance (CMR) to investigate the effects of tafamidis on patients with hereditary A97S ATTR-CM.

**Methods:**

We retrospectively analyzed a prospective cohort of ATTR-CM patients, including 14 with hereditary A97S ATTR-CM and 17 healthy controls with baseline CMR data. All ATTR-CM patients received tafamidis treatment and received CMR with extracellular volume (ECV) at baseline and after 1 year of follow-up.

**Results:**

Baseline N-terminal pro-B-type natriuretic peptide, left ventricular (LV) mass, LV ejection fraction, global radial, circumferential and longitudinal strain, T1 mapping and ECV were significantly worse in the patients with ATTR-CM compared with the healthy controls. After 1 year of tafamidis treatment, ECV decreased from 51.5 ± 8.9% to 49.0 ± 9.4% (P = 0.041), however there were no significant changes in LV mass, LV ejection fraction, global radial strain, global circumferential strain, global longitudinal strain and T1 mapping.

**Conclusions:**

After a one-year treatment period, tafamidis exhibited subtle but statistically significant reductions in ECV, potentially indicating a decrease in amyloid deposition among patients diagnosed with hereditary A97S ATTR-CM.

## Background

Transthyretin amyloid cardiomyopathy (ATTR-CM) is caused by the accumulation of misfolded transthyretin fibrils in the myocardium which leads to heart failure [1, 2]. ATTR-CM is a progressive and life-threatening disease. There are two types of ATTR-CM, a hereditary form (ATTRv) and a wild-type form (ATTRwt). These two types of ATTR-CM have variable clinical presentations, but both have poor long-term outcomes [1, 3]. Tafamidis is an effective treatment for ATTR-CM which can stabilize transthyretin and consequently decrease amyloid formation in these patients [4]. In the Transthyretin Amyloidosis Cardiomyopathy Clinical Trial (ATTR-ACT trial), tafamidis was associated with a decrease in disease progression, all-cause mortality and cardiovascular-related hospitalizations in ATTR-CM patients [5].

Currently, there is no widely accepted tool to monitor the treatment response to tafamidis in ATTR-CM patients [6]. Although serum biomarkers, echocardiography, nuclear studies and cardiac magnetic resonance (CMR) have all been used to investigate the clinical response to tafamidis in patients with ATTR CM, [7] the results have been inconsistent. There is significant variation in the extent of cardiac involvement associated with different transthyretin variants [8]. In Taiwan, the most common type of ATTRv is A97S (p.Ala117Ser). A substantial proportion, up to 82.1% of patients with hereditary A97S ATTR amyloidosis experience cardiac involvement [9]. Unfortunately, few studies have investigated the effects of tafamidis on cardiac remodeling in patients with hereditary A97S ATTR-CM, and this genetic variant was not enrolled in the ATTR-ACT trial [5].

CMR has a unique role in monitoring disease status and treatment response in patients with ATTR-CM, as it can be used to investigate not only the structures of the heart but also tissue characterization including amyloid deposition and fibrosis [10]. CMR techniques are commonly used to detect amyloid deposition, including the patterns of late gadolinium enhancement (LGE), native T1 mapping and extracellular volume (ECV) measurement [11]. ECV is used to demonstrate the percentage of extracellular space, which suggests the degree of amyloid deposition in patients with ATTR-CM [7, 12]. In this study, we aimed to use CMR to investigate the treatment effects of tafamidis on cardiac remodeling in patients with hereditary A97S ATTR-CM.

## Materials and methods

### Study design and subjects

We retrospectively analyzed a prospective cohort of patients with ATTR-CM receiving tafamidis (61 mg/day) treatment at National Taiwan University Hospital. The inclusion criteria were patients: (1) with heart failure symptoms; (2) in whom the diagnosis of ATTR-CM was confirmed by Tc99m-pyrophosphate (PYP) scintigraphy and light chain disease was excluded [13, 14]; (3) with the A97S mutation detected by either Sanger sequencing or restriction enzyme analysis specific for the A97S transthyretin mutation [9, 15]; and (4) who completed 12 months of tafamidis treatment; (5) who underwent CMR at baseline and after 12 months of treatment. The exclusion criteria were patients: (1) who died or were lost to follow-up within 12 months; (2) had poor drug compliance or could not tolerate tafamidis; and (3) had ATTRwt or non-A97S hereditary ATTR-CM. Healthy subjects who underwent CMR studies at National Taiwan University Hospital were also enrolled as controls. All patients provided informed consent before enrollment. This study was approved by the Institutional Review Board of National Taiwan University Hospital.

### Echocardiography

All study subjects received transthoracic echocardiography at enrollment using an IE33 system (Philips Medical Systems; Andover, Massachusetts, USA). The echocardiographic measurements were performed in accordance with the recommendations of the American Society of Echocardiography [16].

### CMR imaging acquisition

CMR was performed on a 1.5-T Magnetom Aera (Siemens Healthcare, Erlangen, Germany) with a 30-channel cardiac coil array. Myocardial T1 mapping was performed with an electrocardiography (ECG)-triggered modified Look-Locker inversion recovery (MOLLI) pulse sequence before and 10 min after.

0.15 mmol/kg intravenous administration of a gadolinium-based contrast agent (Dotarem, Guerbet, France). The MOLLI protocol used a 5(3)3 sampling scheme for native T1 mapping, and a 4(1)3(1)2 sampling scheme for postcontrast T1 mapping. The scan parameters were as follows: TE/TR 1.14/2.7 ms; flip angle 35°; bandwidth.

977 Hz/Px; minimum TI 125–150 ms; TI increment 80 ms; pixel-spacing 1.36 × 1.36 mm2; slice thickness 8 mm; iPAT factor (GRAPPA) 2.

Cine MRI was performed using a segmented balanced steady-state gradient echo pulse sequence with a retrospective ECG R-wave trigger. The scan parameters were as follows: TE/TR 1.6/3.0 ms; flip angle 50–70°; bandwidth 975 Hz/Px; pixel-spacing 1.25 × 1.25 mm2; slice thickness 8 mm; gap 2 mm; iPAT factor (GRAPPA) 2. A total of 10–12 short axis slices were obtained, depending on cardiac size. Thirty cardiac phases were acquired for each level. After postcontrast T1 acquisition, LGE images were acquired using an ECG-triggered phase-sensitive inversion recovery prepared segmented fast gradient-echo pulse sequence to identify focal fibrosis or scarring.

### CMR imaging analysis

The left ventricular (LV) function, myocardial strain and ECV were analyzed offline with commercial postprocessing software (cvi42, Circle Cardiovascular imaging, Calgary, AB, Canada). Epicardial and endocardial contours of the LV were determined at each slice level on cine images, and the area enclosed by each contour was computed for LV function and mass analysis,

LV volumes were then determined using the Simpson rule. End-diastolic volume (EDV) and end-systolic volume (ESV) of the LV were measured from the volume-time curve for the maximal and minimal values. LV mass was computed as the difference between LV epicardial volume at end-diastole and EDV multiplied by the density of the myocardium (1.05 g/ml). LV volumes and mass indexed to body surface area were also measured from EDV, ESV, and LV mass divided by body surface area. Myocardial strain was analyzed on cine images using a tissue tracking technique [17]. We used three long-axis (two-chamber, three-chamber, and four-chamber views) and three short-axis (basal, middle, and apical levels) cine images to measure the peak global systolic radial, circumferential, and longitudinal LV strain. The epi- and endocardial contours were automatically propagated on the end-diastolic images by the software throughout the cardiac cycle generating myocardial strain.

Myocardial ECV was measured from native and postcontrast T1 maps using a region-based method [18]. The regions of interest in the blood and myocardium of the LV were drawn in the central areas of the LV cavity and septal myocardium on the T1-mapping image. If the septal myocardium showed regional hyperenhancement on the LGE images, the region of interest in the myocardium was redrawn in other unenhanced myocardial regions. The average T1 values of the segmented regions of interest were then computed. After subtracting the pre-contrast values from the post-contrast values, the changes in the relaxation rate (1/T1) in the blood and myocardium were obtained. Myocardial ECV values were calculated using the ratio of the change in relaxation rate in the myocardium to that in the blood and multiplied by (1-hematocrit).

### Statistical analysis

Data were expressed as mean ± standard deviation for all continuous variables and number (%) for categorical variables. Comparisons of continuous data between the ATTR-CM patients and controls were performed using the Mann-Whitney U test. Differences between proportions were analyzed using the chi-square test or Fisher’s exact test. The area under the curve (AUC) was used to assess the discriminatory power of the model to predict ATTR-CM. Comparisons of data between baseline and after tafamidis treatment were performed using the Wilcoxon test. All statistical analyses were performed using SPSS for Windows, version 25.0 (SPSS Inc, Chicago, IL).

## Results

### Study subjects

We enrolled 14 patients with hereditary A97S ATTR-CM and 17 healthy control subjects. In our cohort of patients with hereditary A97S ATTR-CM, 15 individuals underwent baseline CMR, while 14 patients received follow-up CMR examinations at the 1 year. Tafamidis was well-tolerated by all patients, and no instances of mortality or loss of follow-up were observed among the enrolled participants. The baseline characteristics were similar between the two groups. One patient in this cohort was reported previously [19]. All these patients also had documented neuropathy and were followed by neurologist. More patients with ATTR-CM had worse New York Heart Association (NYHA) functional class (ATTR-CM: 7% functional class I, 86% functional class II, and 7% functional class III; controls: 100% functional class I, P < 0.001) and significantly higher N-terminal pro-B-type natriuretic peptide (NT-proBNP, 2078.1 ± 3886.3 to 90.2 ± 73.1 pg/ml, P < 0.001). The ATTR-CM patients also had significantly thicker LV anterior and posterior wall thickness as measured by echocardiography (Table 1).

**Table 1:** Baseline characteristics

	ATTR-CM (N = 14)	Control (N = 17)	P value
Age, year	62.1 ± 4.9	60.7 ± 15.4	0.914
Male	13 (93%)	11 (65%)	0.062
Body height, cm	166.6 ± 6.6	166.5 ± 9.2	0.804
Body weight, kg	57.9 ± 11.0	69.6 ± 12.0	0.012
Body mass index, kg/m^2^	20.8 ± 3.6	25.1 ± 3.8	0.006
Diabetes Mellitus	0 (0%)	3 (18%)	0.232
Hypertension	1 (7%)	5 (3%)	0.185
Coronary artery disease	2 (14%)	5 (29%)	0.412
Atrial fibrillation	1 (7%)	0 (0%)	0.452
Pacemaker use	2 (14%)	0 (0%)	0.196
NYHA Fc I	1 (7%)	17 (100%)	< 0.001
NYHA Fc II	12 (86%)	0 (0%)	
NYHA Fc III	1 (7%)	0 (0%)	
**Biochemistry**			
Creatinine, mg/dL	0.8 ± 0.2	0.9 ± 0.4	0.310
Triglyceride, mg/dL	74.5 ± 27.0	109.9 ± 38.4	0.015
Total cholesterol, mg/dL	173.9 ± 31.6	188.1 ± 38.6	0.403
Fasting glucose, mg/dL	83.5 ± 15.0	100.1 ± 15.5	0.003
Albumin, g/dL	4.1 ± 0.2	-	
Prealbumin*, mg/dL	27.1 ± 9.1	-	
NT-ProBNP, pg/dL	2078.1 ± 3886.3	90.2 ± 73.1	< 0.001
LogNT-proBNP	3.0 ± 0.5	1.8 ± 0.4	< 0.001
**Echocardiography**			
IVSD, mm	15.4 ± 2.5	12.0 ± 1.4	0.001
LVPWD, mm	14.8 ± 2.1	9.9 ± 1.2	< 0.001
LVEDD, mm	45.2 ± 3.7	49.4 ± 4.2	0.029
LVESD, mm	31.6 ± 4.8	30.8 ± 5.2	0.616
LVEF, %	56.6 ± 11.7	66.4 ± 9.7	0.238
LA diameter, mm	40.5 ± 4.5	40.4 ± 3.6	0.714

*Prealbumin only available in 6 patients (5 in ECV improved group and 1 in ECV non-improved group)

**Abbreviation**: NYHA = New York Heart Association functional classification; NT-ProBNP = N-terminal pro-B-type natriuretic peptide; IVSD = interventricular septal diameter; LVPWD = Left ventricular posterior wall diameter; LVEDD = left ventricular end-diastolic diameter; LVESD = left ventricle end-systolic diameter; LVEF = left ventricular ejection fraction; LA diameter = left atrial diameter

### CMR imaging analysis

**Fig. 1:**
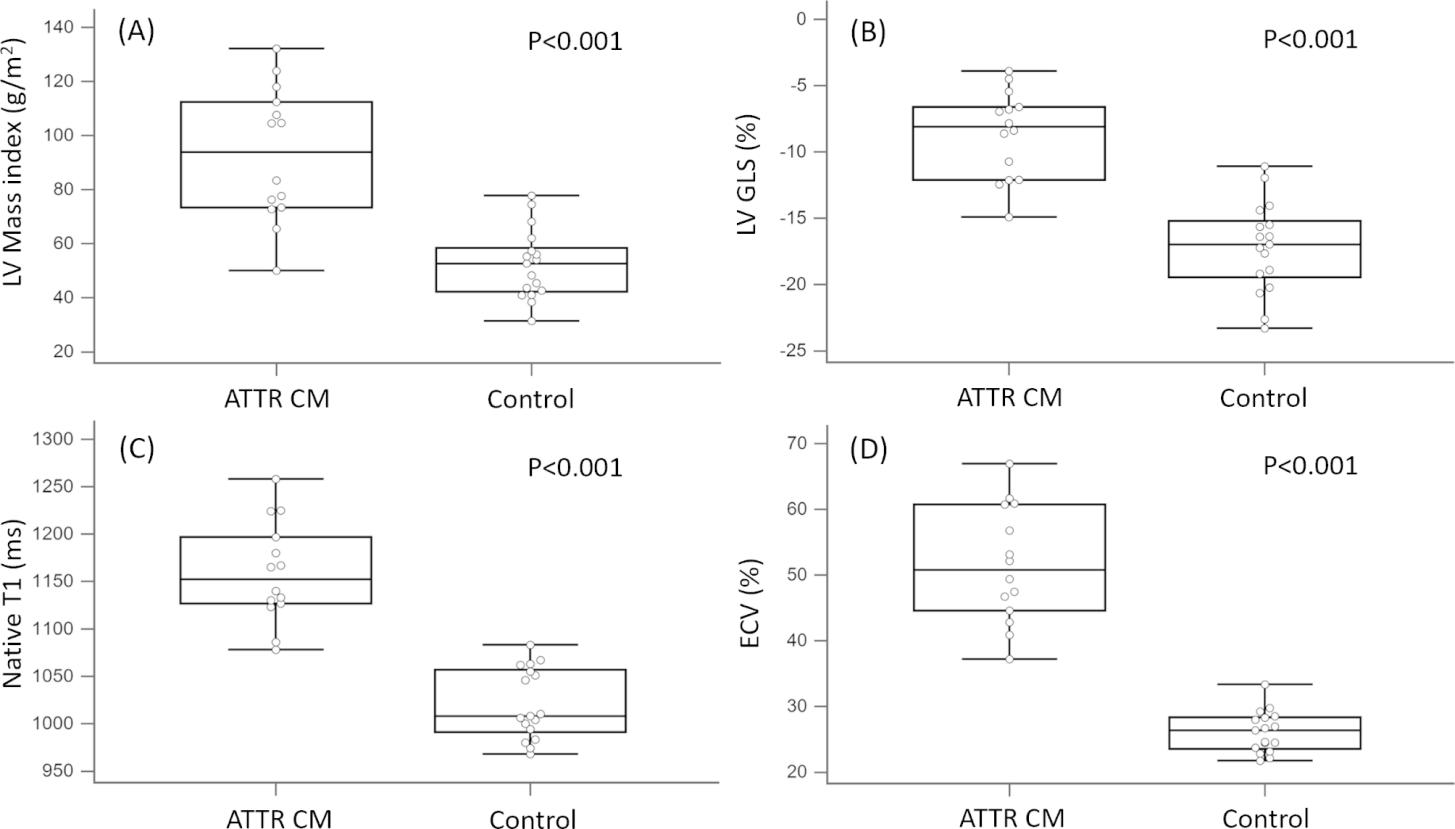
The CMR parameters in patients with ATTR-CM and healthy control

CMR studies showed a significantly lower LV ejection fraction (LVEF) in the patients with ATTR-CM compared with the controls (54.1 ± 9.5% vs. 69.3 ± 10.1%, P < 0.001). The LV end-systolic volume index (LVESVi, 32.3 ± 12.0 vs. 19.9 ± 10.9 ml/m^2^, P = 0.001) and LV mass index (LVMi, 93.0 ± 24.8 vs. 52.3 ± 12.9, P < 0.001) were significantly higher in the patients with ATTR-CM than in the controls. The patients with ATTR-CM had significantly lower global radial strain (GRS, 19.2 ± 8.9 to 36.3 ± 7.7, P < 0.001) and significantly higher global circumferential strain (GCS, -12.6 ± 4.3 to -19.7 ± 2.7, P < 0.001) and global longitudinal strain (GLS, -8.7 ± 3.3 to -17.2 ± 3.4, P < 0.001) compared to the controls. Native T1 mapping (1159.5 ± 53.0 to 1020.8 ± 37.3 ms, P < 0.001) and ECV (51.5 ± 8.9% to 26.1 ± 3.1%, P < 0.001) were significantly higher in the ATTR-CM patients (Table 2; Fig. 1).

**Table 2:** Cardiac MRI parameters

	Total Cohort (N = 14)	Control (N = 17)	P value
Cardiac MRI			
LVEF, %	54.1 ± 9.5	69.3 ± 10.1	< 0.001
LVEDVi, ml/m^2^	69.3 ± 16.7	62.1 ± 15.2	0.109
LVESVi, ml/m^2^	32.3 ± 12.0	19.9 ± 10.9	0.001
LVMi, g/m^2^	93.0 ± 24.8	52.3 ± 12.9	< 0.001
GRS, %	19.2 ± 8.9	36.3 ± 7.7	< 0.001
GCS, %	-12.6 ± 4.3	-19.7 ± 2.7	< 0.001
GLS, %	-8.7 ± 3.3	-17.2 ± 3.4	< 0.001
Native T1 Mapping, ms	1159.5 ± 53.0	1020.8 ± 37.3	< 0.001
ECV, %	51.5 ± 8.9	26.1 ± 3.1	< 0.001

**Abbreviations**: LVEF = left ventricular ejection fraction; LVEDVi = LV end-diastolic volume index; LVESVi = LV end-systolic volume index; LVMi = LV mass index; GRS = global radial strain; GCS = global circumferential strain; GLS = global longitudinal strain; ECV = Extracellular volume

### Comparisons of CMR imaging parameters to differentiate the patients with ATTR-CM from the controls

Among all CMR imaging parameters, ECV had the highest AUC to predict the presence of ATTR-CM in receiver operating characteristic (ROC) curve analysis (AUC: 0.982). The AUCs of all CMR imaging parameters including LVEF, LV end-diastolic volume index (LVEDVi), LVESVi, LVMi, GRS, GCS, GLS, native T1 mapping and ECV were 0.857, 0.669, 0.824, 0.890, 0.879, 0.860, 0.923, 0.974 and 0.982, respectively.

### Treatment response after 1 year of tafamidis treatment

**Fig. 2:**
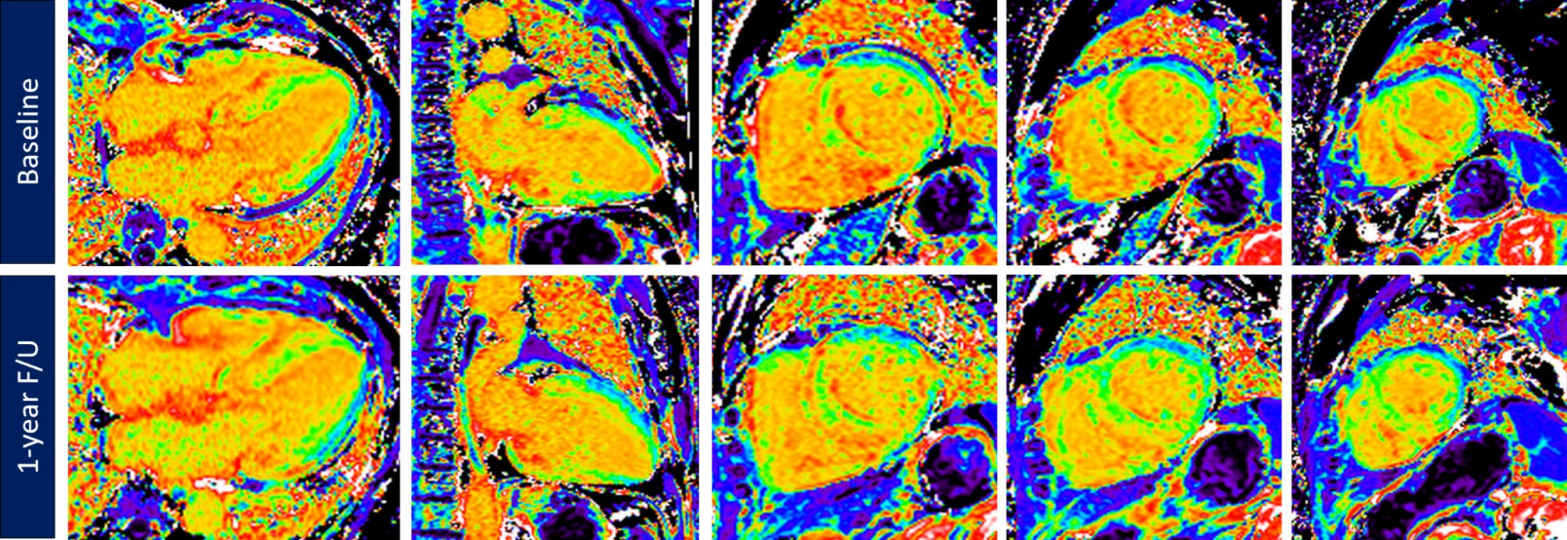
CMR-derived ECV maps before and after tafamidis treatment CMR-derived ECV maps at baseline (top raw) and 1-year follow-up after tafamidis treatment (bottom raw)

**Fig. 3:**
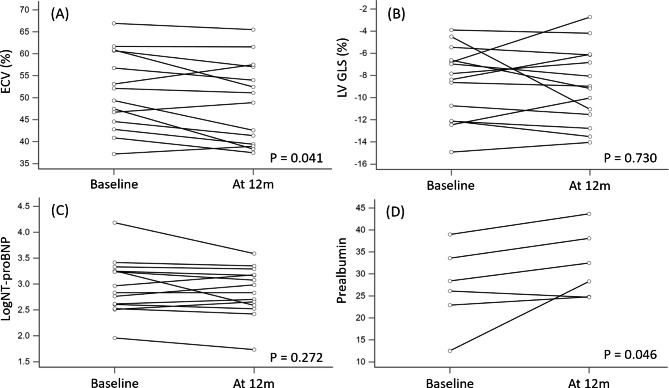
CMR parameters, NT-proBNP and prealbumin before and after tafamidis treatment (**A**) ECV, (**B**) LV GLS, (**C**) LogNT-proBNP, and (**D**) ECV before and after tafamidis treatment

After 1 year of tafamidis treatment, 21% of the patients had improved NYHA functional status, and 79% had no significant change in functional status (P = 0.083). Prealbumin level, which is known as transthyretin (available in 6 patients), significantly improved after tafamidis treatment (27.1 ± 9.1 to 32.0 ± 7.7 mg/dL, P = 0.046). NT-proBNP level improved from 2078.1 ± 3886.3 to 1133.4 ± 1032.6 pg/ml but did not reach statistical significance (P = 0.221). Of the CMR imaging parameters, LVEF, LVEDVi, LVESVi, LVMi, CRS, GCS, GLS and native T1 mapping remained stationary after tafamidis treatment. There was a subtle but statistically significant decreased of ECV after 1 year of tafamidis treatment (51.5 ± 8.9 to 49.0 ± 9.4, P = 0.041) (Figs. 2 and 3; Table 3).

**Table 3:** Treatment response after 1-year Tafamidis treatment

	Pre Tafamidis (N = 14)	12 m Tafamidis (N = 14)	P value
**Functional status**			
NYHA Fc I	1 (7%)	3 (21%)	0.083
NYHA Fc II	12 (86%)	11 (79%)	
NYHA Fc II	1 (7%)	0	
Improved: 3 (21%) Stationary: 11 (79%) Worse: 0 (0%)			
**Biochemistry**			
Creatinine, mg/dL	0.76 ± 0.21	0.70 ± 0.17	0.024
Triglyceride, mg/dL	74.5 ± 27.0	76.6 ± 38.8	0.859
Total cholesterol, mg/dL	173.9 ± 31.6	176.0 ± 37.8	0.610
Fasting blood glucose, mg/dL	83.5 ± 15.0	88.9 ± 23.9	0.834
Albumin, g/dL	4.1 ± 0.2	7.9 ± 12.3	0.833
Prealbumin*, mg/dL	27.1 ± 9.1	32.0 ± 7.7	0.046
Log NT-ProBNP	3.0 ± 0.54	2.9 ± 0.47	0.272
NT-ProBNP, pg/mL	2078.1 ± 3886.3	1133.4 ± 1032.6	0.221
**Echocardiography**			
IVSD, mm	15.4 ± 2.5	15.4 ± 2.8	1.000
LVPWD, mm	14.8 ± 2.1	14.2 ± 2.5	0.119
LVEDD, mm	45.2 ± 3.7	45.0 ± 4.5	0.936
LVESD, mm	31.6 ± 4.8	30.9 ± 6.7	0.550
LVEF, %	56.6 ± 11.7	58.8 ± 15.1	0.594
LA diameter, mm	40.5 ± 4.5	40.5 ± 6.1	0.906
**Cardiac MRI**			
LVEF, %	54.1 ± 9.5	53.1 ± 12.5	0.683
LVEDVi, ml/m^2^	69.3 ± 16.7	76.2 ± 22.3	0.177
LVESVi, ml/m^2^	32.3 ± 12.0	36.4 ± 15.4	0.551
LVMi, g/m^2^	93.0 ± 24.8	97.7 ± 31.2	0.331
GRS, %	19.2 ± 8.9	18.8 ± 9.0	0.433
GCS, %	-12.6 ± 4.3	-12.3 ± 4.5	0.363
GLS, %	-8.7 ± 3.3	-8.9 ± 3.5	0.730
Native T1 Mapping, ms	1159.5 ± 53.0	1157.4 ± 68.2	0.975
ECV, %	51.5 ± 8.9	49.0 ± 9.4	0.041

* Prealbumin only available in 6 patients

**Abbreviations**: IVSD = interventricular septal diameter; LVPWD = Left ventricular posterior wall diameter; LVEDD = left ventricular end-diastolic diameter; LVESD = left ventricle end-systolic diameter; LVEF = left ventricular ejection fraction; LA diameter = left atrial diameter; LVEF = left ventricular ejection fraction; LVEDVi = LV end-diastolic volume index; LVESVi = LV end-systolic volume index; LVMi = LV mass index; GRS = global radial strain; GCS = global circumferential strain; GLS = global longitudinal strain; ECV = Extracellular volume

## Discussion

This study is the first to demonstrate the potentially reversible effects of tafamidis on cardiac amyloid deposition in patients with A97S hereditary ATTR-CM. In the ATTR-ACT trial, tafamidis was shown to decrease all-cause mortality and cardiovascular-related hospitalizations [5]. However, the effects of tafamidis on cardiac remolding and amyloid deposition are still unknown.

ATTR-CM is a progressive and fatal disease which results from the accumulation of misfolded transthyretin in the myocardium. Tafamidis can stabilize transthyretin and theoretically slow disease progression. In the ATTR-ACT trial, tafamidis was shown to significantly improve survival and functional status of patients with ATTR-CM [5]. However, the effects of tafamidis on cardiac structural and functional changes are still under investigation. Several studies using different imaging modalities have demonstrated that tafamidis can slow the progression of cardiac remodeling [20–23]. Most of these studies used echocardiography to measure LV mass and analyze strain, and they showed that tafamidis could slow the progression of cardiac structural and functional changes [21, 22]. Several case reports have demonstrated that tafamidis can reverse cardiac remodeling in patients with ATTR-CM [24–26]. In the current study, cardiac structural and strain analysis measured using CMR remained stationary after 1 year of tafamidis treatment, suggesting that tafamidis can slow disease progression. These results are consistent with a previous study [23].

Rettl et al. were the first to report that tafamidis could delay myocardial amyloid progression in patients with ATTRwt using CMR. In addition, ECV remained stationary (47.5–47.7%, P = 0.861) in the patients who received daily 61 mg tafamidis, but deteriorated in treatment-naïve patients (49.3–54.6%, P = 0.023) [23]. Chamling et al. also demonstrated similar results in the ATTRwt patients, in whom LVMi, GLS and ECV remained stationary after tafamidis treatment. After 12 ± 3 months of tafamidis treatment, ECV decreased from 57 to 54% (P = 0.19) [27]. Both studies investigated the effects of tafamidis on the ATTRwt patients. In contrast, we investigated hereditary A97S ATTR-CM patients in the present study. Our findings revealed a subtle but statistically significant improvement in ECV (51.5 ± 8.9% to 49.0 ± 9.4%, P = 0.041) following one year of tafamidis treatment. Since tafamidis is the transthyretin stabilizer, tafamidis has demonstrated greater efficacy in patients at earlier stages of the disease. In our study, the majority of patients (93%) presented with NYHA function class I to II. This observation may provide a potential explanation for the effectiveness of tafamidis observed in this study. The genetic variant is the other potential explanation. A97S is the most common ATTR variant in Taiwan, however it has been less frequently investigated in previous clinical trials. A97S hereditary ATTR amyloidosis is relatively late onset. Majority these patients have cardiac involvement, and refractory heart failure is the major cause of death [9, 28].

Other novel disease-modifying therapies can also treat ATTR-CM. Two novel TTR gene silencers including the small interfering RNA patisiran and the antisense oligonucleotide inotersen have been investigated in patients with ATTR-CM [29]. Fontana et al. demonstrated that patisiran could reduce the progression of amyloid deposition, and that the patients who received patisiran had stationary ECV values compared with historical controls [7]. Data on the effects of inotersen using CMR are still limited. In the current study, we used CMR to demonstrate the excellent effects of tafamidis on slowing the progression of cardiac remodeling and the potential ability to reverse it in patients with A97S hereditary ATTR-CM. Our results suggest that amyloid deposition may not be an irreversible process, but rather that it is dynamic and reversible. However, this study only enrolled A97S hereditary ATTR-CM patients and the number of patients was small. Therefore, the results should be interpreted carefully.

CMR is a valuable and non-invasive diagnostic tool for cardiomyopathy which can assess tissue characterization, cardiac structure and function [10]. It is practically useful in patients with inconclusive results on 99mTc-PYP scintigraphy. In these cases, CMR can help the clinician to detect amyloid deposition and also guide the need for an endomyocardial biopsy. There are many different imaging techniques in CMR, including traditional cine CMR, late gadolinium enhancement, native T1 mapping (non-contrast) and ECV (with contrast) measurement [30]. The ECV is usually ≥ 40% in patients with ATTR-CM, which is a useful feature to differentiate it from other cardiomyopathies [31, 32]. In the current study, the ECV had the greatest power to differentiate the ATTR-CM patients from the healthy controls (AUC: 0.982). In addition, the ECV improved after tafamidis treatment, which suggests the potential role of ECV to monitor treatment response.

Notably, although no statistically significant improvement in NYHA functional status was observed in this study, a noticeable trend of functional status improvement was detected. The duration of our follow-up period was relatively short compared to the ATTR-ACT study, where significant improvement in clinical endpoints was observed after 30 months, with a noticeable trend towards improvement at around 15 months [5]. In contrast, our study had a follow-up period of only 12 months. Furthermore, due to the small sample size, our study may lack statistical power to answer this question.

### Limitations

There are several limitations to this study. First, this was a small, single-arm, prospective cohort study. Further larger studies with a control group are needed to confirmed our findings. However, the results of the current study are still valuable, since data on the treatment effects of tafamidis in patients with A97S ATTR-CM are still very limited. Second, not all of the patients had complete prealbumin data, which may have affected the results. Third, this is a prospective cohort study with retrospective analysis. Potential selection bias may have interfered with the results, and thus they should be interpreted carefully. Fourth, the follow-up period was relatively short compared with previous randomized control trial [5]. However, this study provides valuable information about the early change of ECV after tafamidis treatment in patients with A97S ATTR-CM. Fifth, the enrollment of predominantly male patients in this study introduced the possibility of gender bias, which could potentially impact the results of the study.

## Conclusions

In conclusion, tafamidis can slow the progression of cardiac remodeling in patients with A97S hereditary ATTR-CM. In addition, tafamidis decreased ECV in our patients with hereditary A97S ATTR-CM, suggesting it might lower amyloid deposition. Our findings suggest the potential role of tafamidis to reverse cardiac remodeling in patients with A97S hereditary ATTR-CM.

## Data Availability

The patient-level data of this study is not publicly available; however, it can be obtained from the authors upon reasonable request and with the permission of National Taiwan University Hospital.

## List of Abbreviations

ATTR-CM: Transthyretin amyloid cardiomyopathy
ATTRv: Hereditary ATTR
ATTRwt: Wild-type ATTR
ATTR-ACT trial: Transthyretin Amyloidosis Cardiomyopathy Clinical Trial
AUC: Area under the curve
CMR: Cardiac magnetic resonance
ECV: Extracellular volume
ECG: Electrocardiography
EDV: End-diastolic volume
ESV: End-systolic volume
GRS: Global radial strain
GCS: Global circumferential strain
GLS: Global longitudinal strain
LVESVi: LV end-systolic volume index
LVEF: LV ejection fraction
LVEDVi: LV end-diastolic volume index
LVMi: LV mass index
MOLLI: Modified Look-Locker inversion recovery
ROC: Receiver operating characteristic
NT-proBNP: N-terminal pro-B-type natriuretic peptide
